# Next-Generation Immunotherapy for Hepatocellular Carcinoma: Mechanisms of Resistance and Novel Treatment Approaches

**DOI:** 10.3390/cancers17020236

**Published:** 2025-01-13

**Authors:** Shabnam Eghbali, Thatcher Ross Heumann

**Affiliations:** 1 Division of Internal Medicine, Department of Medicine, Vanderbilt University Medical Center, Nashville, TN 37232, USA; 2 Division of Hematology and Oncology, Department of Medicine, Vanderbilt University Medical Center, Nashville, TN 37232, USA; 3 Vanderbilt Ingram Cancer Center, Nashville, TN 37232, USA

**Keywords:** immunotherapy, hepatocellular carcinoma, angiogenesis, glypican-3, Wnt/β-catenin, bispecific antibodies, CAR-T cell therapy, locoregional therapy, neoadjuvant, cancer vaccine

## Abstract

Hepatocellular carcinoma (HCC) is a leading cause of cancer-related mortality worldwide, and, with only 15–20% of HCC patients being suitable for potentially curative treatments, the vast majority of patients with HCC ultimately require systemic therapy. For decades, the choice of effective systemic therapy for HCC remained sparse. In recent years, after the combination of atezolizumab and bevacizumab demonstrated superior overall survival over the first-line standard, sorafenib, there has been a major therapeutic paradigm shift to immunotherapy-based regimens for HCC. While representing a great leap forward for the treatment of this cancer, the reality is that less than one-third of patients achieve an objective response to immune checkpoint inhibitor-based therapy, so there remains a significant clinical need for further therapeutic optimization. In this review, we provide an overview of the current landscape of immunotherapy for unresectable HCC and delve into the tumor intrinsic and extrinsic mechanisms of resistance to established immunotherapies with a focus on novel therapeutic targets with strong translational potential. Following this, we spotlight emerging immunotherapy approaches and notable clinical trials aiming to optimize immunotherapy efficacy in HCC that include novel immune checkpoint inhibitors, tumor microenvironment modulators, targeted delivery systems, and locoregional interventions.

## Simple Summary

In recent years, there has been a paradigm shift in first-line treatment for unresectable hepatocellular carcinoma (HCC) from multitargeted tyrosine kinase inhibitors to immune checkpoint inhibitor-based therapies. Despite the unprecedented improvement in clinical outcomes, responses to these therapies are still only observed in a minority of HCC patients. In this review, we discuss the tumor intrinsic and extrinsic mechanisms of resistance to standard immune checkpoint inhibitors, explore novel approaches to optimize immunotherapy-based regimens, and highlight notable clinical trials that have the potential to change future standard care for HCC.

## 1. Introduction

Hepatocellular carcinoma (HCC) constitutes up to 85% of primary liver cancer cases, with rising incidence and mortality worldwide. Considerable challenges exist in the clinical management of HCC. Only 15–20% of patients are diagnosed at an early stage such that they may be suitable for potentially curative treatments, with the vast majority of patients diagnosed with unresectable disease that ultimately requires systemic therapy. Tyrosine kinase inhibitors (TKIs), such as sorafenib and lenvatinib, have been the mainstay of HCC treatment for many years. In 2020, a treatment paradigm shift occurred when atezolizumab, an anti-PD-L1 monoclonal antibody (mAb), and bevacizumab, an anti-VEGF-A mAb, demonstrated superior overall survival when compared to then first-line standard, sorafenib [1]. Since then, the standard of care for first-line advanced, unresectable HCC has shifted to immune checkpoint inhibitor (ICI)-based therapy, with ICI-based combination therapies now dominating clinical trials for HCC.

However, despite the evolution of current standard HCC systemic therapy, less than one-third of patients with HCC achieve an objective response to ICI-based therapy [2,3]. Thus, identifying predictive biomarkers for ICI response, mechanisms of ICI-resistance, and novel therapeutic targets to overcome these resistance mechanisms are all paramount to unlocking the full potential of immunotherapy in HCC. In this review, we will do the following: (1) provide an overview of the current landscape of immunotherapy for unresectable HCC; (2) delve into mechanisms of resistance to established immunotherapies, with a focus on mechanisms with translational and therapeutic implications; and (3) discuss emerging, novel immunotherapy approaches to optimize immunotherapy efficacy in HCC.

## 2. Current Immunotherapy for Advanced Unresectable HCC

Immune checkpoint inhibitors (ICIs) that target programmed cell death protein-1 (PD-1) or its ligand, programmed cell death-ligand 1 (PD-L1), and cytotoxic T lymphocyte antigen 4 (CTLA-4), have become the backbone of cancer therapy for a multitude of solid tumors. The immune tolerogenic trait of the liver, coupled with the immunosuppressive microenvironment in HCC, may contribute to antitumor immunity in HCC, making ICI a potentially compelling therapeutic approach to treating HCC. In addition to its ability to evade the immune system, HCC is also an angiogenic tumor, enabling multitargeted TKIs, like sorafenib [4] and lenvatinib [5], that target angiogenesis to dominate systemic therapy for HCC for over a decade. The first-line paradigm shifted in 2020 with the phase 3 IMBRAVE150 study, which assessed combination therapy with atezolizumab, an anti-PD-1 mAb, plus bevacizumab, an anti-vascular endothelial growth factor A (anti-VEGF-A) mAb, versus the then-first-line sorafenib [1]. In this study (n = 501), patients who received first-line atezolizumab plus bevacizumab demonstrated superior overall survival (OS) (median OS (mOS) 19.2 mo [atezolizumab-bevacizumab] versus 13.4 mo [sorafenib], HR 0.66) and objective response rate (ORR) (30% [atezolizumab-bevacizumab] versus 5% [sorafenib]) (Table 1). This established atezolizumab plus bevacizumab as the standard of care for first-line therapy in patients eligible for treatment with immunotherapy and anti-angiogenic therapy. The combination of immunotherapy and tyrosine kinase inhibitors was a natural next step to assess following this, but it came with underwhelming results, as the combination of lenvatinib with pembrolizumab (phase 3 LEAP-002 [6]), and cabozantinib with atezolizumab (phase 3 COSMIC-312 [7]), did not lead to significant survival benefit over their respective TKI-only control arm (Table 1). An exception to this was the positive phase 3 CARES-310 [8] that assessed camrelizumab, an anti-PD-1 mAb, and rivoceranib, a highly selective VEGFR-2 targeted TKI (with a half-life of 9 h compared to 20 days for bevacizumab [9,10]). Patients treated with the combination showed improved OS (mOS 22.1 mo [camrelizumab-rivoceranib] versus 15.2 mo [sorafenib], HR 0.62) (Table 1).

Immune checkpoint inhibitor-exclusive regimens, specifically the combination of CTLA-4 and PD-1/PD-L1 antagonist mAbs, have also shown significant clinical benefits in the first-line setting. The combination of single-dose tremelimumab, an anti-CTLA-4 mAb, and durvalumab, an anti-PD-L1 mAb (together known as STRIDE), became the first immune checkpoint inhibitor-exclusive regimen to show superior OS compared to first-line sorafenib in the phase 3 HIMALAYA trial [3]. Patients who received STRIDE had significantly improved OS (mOS 16.4 mo [STRIDE] versus 13.8 mo [sorafenib], HR 0.78) and ORR (20.1% [STRIDE] versus 5.1% [sorafenib]) compared to those randomized to sorafenib. It should also be noted that durvalumab monotherapy was found to be non-inferior to sorafenib in OS (mOS 16.5 mo [durvalumab] versus 13.77 mo [sorafenib]), HR 0.86) in that same trial (Table 1). The combination of ipilimumab, an anti-CTLA-4 mAb, and nivolumab, an anti-PD-1 mAb, also was found to improve survival over lenvatinib or sorafenib in the first-line phase 3 CHECKMATE 9DW with those treated with the dual checkpoint inhibitor regimen achieving superior OS (mOS 23.7 mo versus 20.6 mo, HR 0.79) and ORR (36% versus 13%) [11] (Table 1). These regimens are particularly attractive options for patients who are considered to be at high risk for anti-angiogenic agents. Immunotherapy combinations clearly have an effect on patients, but the sobering fact remains that only about one in four patients are likely to respond to current standard regimens; thus, further optimization of immunotherapy remains paramount in HCC.

## 3. Mechanisms of Resistance to Conventional Immune Checkpoint Inhibition

Given the widespread use of ICI across multiple solid tumors, several mechanisms of resistance have been described in the literature, often focusing on aberrancies with T cell activation, e.g., impaired antigen recognition, barriers to T cell migration and infiltration, and weakened effector T cell functions [12,13,14]. More recent data [15,16] have highlighted the role of the adaptive immune system and other immunosuppressive elements in the tumor microenvironment (tumor extrinsic), as well as the characteristics of cancer cells themselves (tumor intrinsic) in promoting resistance to ICI. Below, we focus on tumor extrinsic and intrinsic mechanisms that have been characterized in HCC for which there are current translational work and clinical trials ongoing to overcome these mechanisms of resistance.

### 3.1. Tumor Extrinsic—Immunosuppressive Tumor Microenvironment

HCC is composed of a complex tumor microenvironment (TME) consisting of cellular (e.g., immune cells, stromal cells), chemical (e.g., cytokines, chemokines), and physical (e.g., extracellular matrix) components. The etiology of HCC can lead to unique microenvironmental features. In contrast to viral hepatitis, which is characterized by focal, organized inflammatory foci, metabolic dysfunction-associated steatohepatitis (MASH)-induced inflammation is more often associated with dispersed inflammatory infiltrates [17]. PD-1-expressing regulatory T cells are enriched in HBV-related disease, whereas CD8+ T cells and NK cells are most prevalent in non-viral HCC [18]. These CD8+ T cells are governed by metabolic dysregulation, resulting in MHC I-independent cytotoxicity against hepatocytes that drives necrosis and inflammation and loss of tumor surveillance function [19]. Understanding these differences based on the etiology of HCC can provide clues to the varying responses to ICI.

Within the cellular compartment in the TME is an interplay between the innate and adaptive immune systems. HCC commonly develops in the context of chronic liver inflammation, mostly elicited by innate immune activation [17]. Molecular analyses performed on tumor samples from patients enrolled in the IMBRAVE150 study highlight the importance of pre-existing immunity—defined as a high expression of CD274 (PD-L1), T-effector signature (CXCL9, PRF1, GZMB), and intratumoral CD8+ T cell density—in terms of clinical response to ICI [20]. The key cells with major immunosuppressive roles implicated in immune evasion in HCC are extensively discussed in numerous reviews [21,22], thus below we focus on cellular and non-cellular components with current or emerging therapeutic applications.

#### 3.1.1. Tumor-Associated Macrophages (TAMs)

**Figure 1:**
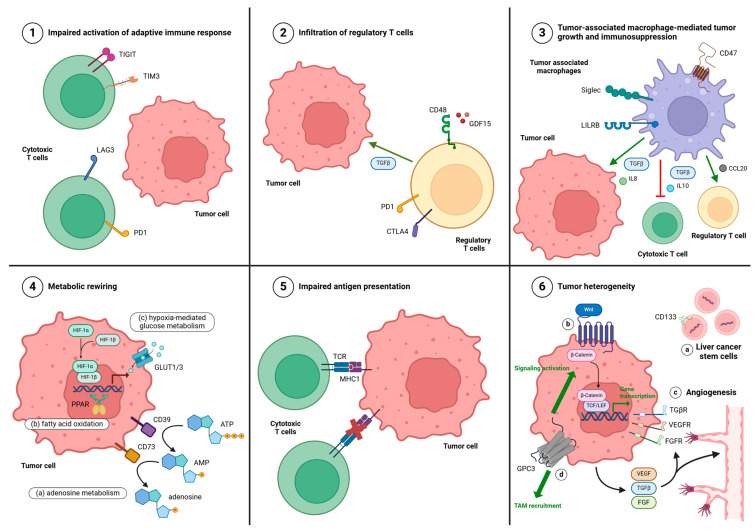
Mechanisms of resistance to immune checkpoint inhibitors. A complex interplay of tumor intrinsic and extrinsic pathways work to facilitate resistance to ICI. (1) Cytotoxic T cells express other checkpoint inhibitors beyond PD1 and CTLA4, such as LAG3, TIM3, and TIGIT, that can contribute to immune evasion. (2) GDF15, through its interaction with CD48, enables the immunosuppressive properties of Tregs and promotes hepatocarcinogenesis via secreted factors such as TGFβ. The adaptive immune system, such as tumor-associated macrophages (3), can facilitate HCC progression through the secretion of factors, e.g., TGFβ, and the expression of receptors, e.g., LILRB, that are pro-tumorigenic. Metabolic rewiring, such as increases in adenosine metabolism (4a), fatty acid oxidation via PPAR (4b), and hypoxia-induced glucose metabolism (4c), can shape an immunosuppressive environment in a way that greatly hinders the efficacy of ICI. Antigen presentation is key to generating the immune response and relies on HLA-1 molecules, which are often aberrantly expressed in HCC, leading to ICI treatment failure (5). Tumor heterogeneity, as reflected in the presence of liver cancer stem cells (6a) that work as an evergreen tool for driving the suppressive activity of immune cells in the TME, the aberrant activation of various cellular signaling networks that have impacts on tumor growth (6b), immune cell evasion, angiogenesis (6c), and the expression of tumor-associated antigens (6d), underpins differences in responses to ICI. This figure was created with www.BioRender.com (accessed on 8 January 2025).

TAMs account for 20–40% of immune cells in HCC [23], and thus play a significant role in facilitating HCC progression through cancer cell reshaping and growth, immunosuppression, angiogenesis, and extracellular remodeling [24] (Figure 1(3)). Lu et al. generated a molecular atlas of the multicellular ecosystem of primary and metastatic HCC using single RNA sequencing that revealed seven TME subtypes of HCC, with the macrophage-dominated and lymphocyte-depleted microenvironments conferring the worst prognosis; these environments have a large population of *MMP9+* TAMs, with PPARγ driving their terminal differentiation and thus HCC progression [25]. TAMs can be polarized into different phenotypes, including tumor-inhibiting M1 macrophages and tumor-promoting M2 macrophages. HCC cells secrete osteopontin, sonic hedgehog, and GP73 that promote the M2 polarization of TAMs [26]. In turn, M2 TAMs secrete a number of factors that create a pro-tumorigenic TME in HCC. Il-8, IL-10, and gal-1 (through its activation of the pro-cancerous mTor-Akt pathway) stimulate tumor proliferation [23,27]. Clever-1 impairs Th1 T cell activation, and thus prevents macrophage inactivation [28], whereas IL-10 and TGFβ inhibit the activation of cytotoxic T cells and NK cells [22]. CCL17, CCL18, CCL20, CCL22, and IGF135 trigger the recruitment of Tregs [22,26]. VEGF, PDGF, IL-17, MMP2, and MMP9 promote HCC cell neovascularization [22]. TGFβ [29] and CCL20 [30,31] also promote epithelial–mesenchymal transition (EMT). S100 calcium binding protein A9 elevates the cancer stem-like ratio [32], and increasing cancer stemness contributes to resistance to therapy. Myeloid immune checkpoint molecules, such as CD47, the signaling lymphocytic activation molecule (SLAM) family, the sialic acid-binding immunoglobulin-type lectin (Siglec) family, and the leukocyte immunoglobulin-like receptor B (LILRB) family [33,34], help mediate inhibitory signals that promote the pro-tumor immune activities of TAMs. In fact, the LILRB family was found to have increased expression in peripheral blood and in tissue from patients with HCC compared to healthy donors [35]; an overexpression of LILRB2 in particular was positively and significantly correlated with poor prognostic features in HCC patients, including poor cell differentiation, larger primary tumor size, and shorter overall survival [36]. Targeting TAMs could be a potentially beneficial way to bridge the innate and adaptive immune systems and to work synergistically with ICI. This could be done by preventing the infiltration of TAMs, reprogramming macrophage phenotypes towards pro-inflammatory and antitumoral M1-types, or inhibiting their tumor-promoting functions via the inhibition of myeloid checkpoint molecules.

#### 3.1.2. Regulatory T Cells (Tregs)

A meta-analysis of patients with HCC demonstrated that a higher infiltration of Tregs indicated lower overall survival and disease-free survival [37]. In hypoxia-high tumors like HCC, Tregs and immunosuppressive myeloid subsets, such as type 2 conventional dendritic cells (cDC2s), were found to be significantly enriched, and the interaction between Tregs and cDC2 led to a loss of antigen presentation on cDC2s, suggesting a unique immunosuppressive pathway [38]. Moreover, in terms of molecular correlates of clinical resistance to atezolizumab and bevacizumab, a low ratio of regulatory T cells to effector T cells was associated with improved PFS and OS [20]. Thus, targeting Tregs and their associated factors, such as cytokines, which promote their recruitment, could help mitigate HCC progression and resistance to ICI. Tregs are commonly recruited by the CCR6-CCL20 pathway and inhibit immune response through CTLA4, CD39/CD73, TGFβ, IL-10, and GDF15/CD48 [39,40], thus serving as potential therapeutic targets. Growth differentiation factor (GDF15) in particular is a member of the TGF superfamily that exerts its immunosuppressive actions in HCC through Tregs, where it binds to the CD48 receptor inhibiting the ERK-Activator protein 1 pathway, causing downstream FOXP3 accumulation and HCC development [40,41]. In a murine model of HCC, the gene ablation of GDF15 slowed tumor growth, prolonged the survival of mice, and decreased GDF15 concentration and Treg cell frequency [41] (Figure 1(2)).

#### 3.1.3. Non-Cellular Components

Disruption of the extracellular matrix (ECM), induction of hypoxia, and metabolic reprogramming all work to influence the functions of the cellular components of the TME, and thus facilitate therapeutic resistance in HCC. 

The ECM is a dynamic component of the TME that responds and adapts to environmental changes. Dysregulation of the ECM in terms of its remodeling and degradation influences growth factor pathways, cytokine signaling, angiogenesis, and interactions with the cellular component of the TME that ultimately promote tumor growth and progression [42]. For example, researchers have identified six ECM-associated genes, namely SPP1, ADAMTS5, MMP1, BSG, LAMA2, and CDH1, that are involved in the destruction of different proteins within the ECM, resulting in increased EMT, and that correlate with poor prognosis in HCC [43]. Moreover, the ECM is able to enrich the population of liver cancer stem cells, which are one of the key contributors to therapy resistance, through direct integrin-mediated interactions, as well as the induction of hypoxia, which activates self-renewal [44].

Intratumoral hypoxia is a crucial feature of all solid tumors. IHC studies revealed significantly higher expression of hypoxic markers, including HIF-1α, glucose transporter type 1 (GLUT1), lactate dehydrogenase A (LDHA), and carbonic anhydrase 9 (CA9) in human HCC tissues when compared to nontumorous liver tissues [45].

Metabolic rewiring under hypoxia contributes to drug resistance. Levels of metabolites, such as glucose, lactate, and adenosine, are altered in the TME such that they collectively help to shape an immunosuppressive environment and greatly hinder the efficacy of ICIs in HCC. Nutrient competition between cancer cells and antitumor immune cells is a common phenomenon in HCC that contributes to ICI resistance [45]. HIF-1 induces the expression of solute carrier family 2 member 1 (SLC2A1) and solute carrier family 2 member 3 (SLC2A3), which encode GLUT1 and GLUT3, respectively, to promote glucose uptake to meet the demand of glucose for the growth of hypoxic cancer cells. An overexpression of GLUT1/3 was associated with poor clinical outcomes, including a more advanced tumor stage, greater tumor burden, higher rate of recurrence, and poor survival in HCC patients [46]. AMP and adenosine are immunosuppressive metabolites. ATP is converted to AMP and adenosine by HIF-induced ectoenzymes, CD39/CD39L1, and CD73, leading to the accumulation of AMP and adenosine under hypoxia (Figure 1(4a)). This accumulation inhibited the differentiation of myeloid-derived suppressor cells (MDSCs), leading to the accumulation of immunosuppressive MDSCs in hypoxic HCC [47].

Another metabolic adaptation is an increase in fatty acid oxidation (FAO), which promotes metastasis and modulates immune cell function, ultimately impacting drug resistance [48]. Peroxisome proliferator-activated receptors (PPARs) are a family of fatty acid ligand-activated transcription factors that regulate genes involved in the regulation of energy homeostasis, including fatty acid oxidation, angiogenesis, and inflammation (Figure 1(4b)). TCGA-based analysis of tumor metabolic gene expression profiles show a high expression of PPAR and thirty other FAO-related genes in HCC compared to other tumor types [49]. Three distinct subtypes have been identified—PPARα, PPARγ, and PPARβ/δ. In total, 40% of patients with HCC resistant to pembrolizumab had high PPARγ induction [50]. One possible explanation that researchers uncovered in immunotherapy-resistant HCC murine models is that tumor cell intrinsic upregulation of PPARγ transcriptionally activated VEGF-A production, which drove MDSC expansion and CD8+ T cell dysfunction [50]. A selective PPARγ antagonist triggered an immune suppressive-to-stimulatory TME conversion and resensitized tumors to anti-PD-L1 therapy in the aforementioned murine models [50].

### 3.2. Tumor Intrinsic–Impaired Antigen Presentation

Antigen presentation plays an integral role in immune response, whereby the antigenic environment is processed by antigen-presenting cells so that they can be recognized by T cells. The CD8+ T cell-dependent killing of cancer cells requires the presentation of tumor antigens by human leukocyte antigen class I (HLA-I) molecules. Several studies have shown that impaired antigen presentation leads to treatment failure with ICIs [51]. Impaired antigen presentation can encompass processes that affect the number or processing of tumor-associated antigens (TAAs) and neoantigens, or the antigen presentation machinery itself, or the recognition of neoantigens by the adaptive immune system. An evaluation of the frequency of genomic biomarkers of ICI response in 755 patients with advanced HCC showed that the median tumor mutation burden (TMB) was four mutations/Mb, with only six (0.8%) patients exhibiting a high TMB, and that there was no significant genomic or TMB differences between responders, progressors, and those with stable disease [52]. More specifically, with the current standard of care, atezolizumab plus bevacizumab, there is no apparent association between TMB and response rate or survival benefit [20]. High TMB is not associated with high levels of immune infiltration [17]. This can be partially explained by the molecular alterations that disrupt antigen-presenting machinery [53] (Figure 1(5)). For example, researchers found that high broad copy-number alteration burdens in HCC can lead to structural losses of genes involved in antigen presentation, leading to immune exclusion [54,55]. Moreover, a loss of HLA alleles, identified in 17% of HCC, hampered the ability of the major histocompatibility complex to present neoantigens [54]. Though CD8+ T cells may need to be present to recognize TAAs and neoantigens, they are not able to mount an effective immune response because they are not efficiently induced, are only detected in 15% of patients with HCC [56], and exist in an innate-like low cytotoxic state [57].

### 3.3. Tumor Intrinsic–Tumor Heterogeneity 

The wide application of next-generation sequencing has given us insight into the tumor heterogeneity that exists among patients with HCC and likely underpins varied responses to ICI. This tumor heterogeneity is likely driven by liver cancer stem cells, the aberrant activation of various cellular signaling networks that have impacts on tumor growth, immune cell evasion, and angiogenesis, and the expression of tumor-associated antigens (TAAs).

Liver cancer stem cells (LCSCs) are defined as cell clusters with surfaces enriched in markers including epithelial cellular adhesion molecule (EpCAM), CD133, CD13, CD90, CD44, CD24, and calcium channel a2d1 subunits [58]. The number of circulating LCSCs positively correlates with advanced, invasive stages of HCC. As discussed above, elements of TME can influence the generation of LCSCs and maintain their stemness. For example, periostin, a secretory protein that plays a crucial role in angiogenesis and is highly expressed in 60% of HCC patients, through a positive feedback loop with TGFβ, can activate AP-2α to transcriptionally induce the expression of CD133, which promotes the stemness transformation of HCC cells [58]. There are several proposed mechanisms through which they are able to mediate resistance to ICI: differentially expressed intracellular and surface markers that contribute to immune evasion; the secretion of cytokines that influence the recruitment, survival, and function of immunosuppressive cells; and related metabolites that modulate the activity of infiltrated immune cells [59]. For example, the secretion of IL-8 stimulates the M2 polarization of TAMs [60], and the secretion of IL-6 strengthens the immunosuppressive functions of MDSCs [61] (Figure 1(6a)).

The Wnt/β-catenin pathway is one of the most characterized and cited cellular signaling pathways in the tumorigenesis and progression of HCC and resistance to therapy [62]. Up to 40% of HCC tumors show constitutive activation of this pathway. Wnt/β-Catenin pathway mutations frequently occur in HCC patients resistant to ICI [63]. In a study exploring genetic biomarkers of responsiveness or resistance, for patients treated with ICI (n = 31), activating alterations of WNT/β-catenin signaling compared to no alterations were associated with lower disease control rate (DCR) (0% versus 53%), shorter mPFS (2.0 months versus 7.4 months), and shorter mOS (9.1 months versus 15.2 months) [64]. One way this pathway is able to mediate ICI resistance is by impairing antigen-specific T cell-mediated antitumor immunity [63]. CTNNB1 mutations and/or the activation of the WNT/β-catenin signaling pathway downregulates the expression of CCL5 and impairs dendritic cell recruitment, thus promoting immune escape and resistance to ICIs in murine models of HCC [65]. Finally, the Wnt/β-catenin pathway has also been implicated in the enrichment of liver cancer stem cells and in maintaining their stemness [66] (Figure 1(6b)).

Increased angiogenesis is one of the defining features of HCC, thus the activation of these signaling pathways, namely transforming growth factor beta (TGFβ), vascular endothelial growth factor (VEGF), and fibroblast growth factor (FGF), often portends a poor prognosis. Increased TGFβ activity may suppress the antitumor response through inhibiting CD4+ T helper cell function, decreasing the differentiation and function of cytotoxic T cells, promoting the pro-tumor TH17 response, and recruiting immune suppressive myeloid cells into the tumor microenvironment [67]. Single-cell RNA sequencing data have identified VEGF as key to reprogramming the TME into an immunosuppressive landscape by operating as a potent immunomodulator, affecting both innate (e.g., TAMs) and adaptive immune cells (e.g., Tregs, effector T cells) [68]. The inhibition of VEGF signaling was found to promote vessel normalization, resulting in improved drug delivery, as well as the potential enhancement of immune cell attachment and extravasation [69]. Aberrant expression of FGF family receptors and ligands promotes HCC proliferation through their impact on angiogenesis, metabolism, and EMT [70,71]. FGFR4 shows the highest expression on hepatocytes, and through its ligands, namely FGF2, FGF8, and FGF19, help drive the aggressive phenotype in HCC [72]. FGF19 amplification was independently associated with shorter survival and a higher risk of recurrence in patients with HCC, and was correlated with poor prognostic factors such as high α-fetoprotein and microvascular invasion [73] (Figure 1(6c)).

Glypican-3 (GPC3) is a membrane protein expressed in 70% of HCC, but rarely expressed in normal liver tissue, making it a key TAA. GPC3 has been linked to HCC tumorigenesis through multiple mechanisms, including the recruitment of TAMs, enhancing glucose metabolism, EMT, apoptosis resistance via the Bax/Bcl-2 pathway, and cellular signaling, like the Wnt-β-catenin pathway [74,75] (Figure 1(6d)). A retrospective biomarker analysis of phase 1b and phase 3 trials with atezolizumab and bevacizumab identified that increased expression of GPC3, AFP, and activated beta-catenin are associated with resistance to PD-1 blockade and, vice versa, decreased levels are associated with increased PFS and OS [20]. Though the role of GPC3 in HCC has not been fully elucidated, the number of associations with various cell signaling and immune-related pathways known to play roles in HCC and its correlation with poor prognosis makes GPC3 a promising biomarker and therapeutic target in GPC3-expressing tumors like HCC.

## 4. Novel Immunotherapy Approaches

Since the approval of atezolizumab and bevacizumab for first-line advanced unresectable HCC, there has been a burst of clinical trials assessing the utilization of immunotherapy across all stages of disease. Below, we focus on emerging strategies for patients with advanced HCC, starting with ICI-based combinations before transitioning to a review of promising new treatment modalities. We end with a brief discussion on the expanded use of ICI in neoadjuvant and adjuvant settings.

### 4.1. ICI-Based Combinations

Most current clinical trials in HCC seek to develop ICI-based doublet or triplet therapy with next-generation ICI or targeted small molecule inhibitors or locoregional therapy.

#### 4.1.1. Targeting Adaptive and Innate Immune Cells

Despite both anti-PD-1 and anti-CTLA4 mAbs forming the basis of ICI, the widespread use of anti-CTLA4 mAb, either as monotherapy or in combination with anti-PD-1/PD-L1, has been limited due to dose-dependent toxicity. ADG126 is a masked anti-CTLA4 mAb targeting a unique epitope of CTLA4 on Tregs and deplete Tregs by antibody-dependent cellular cytotoxicity. The masked nature of this antibody enables it to bind to its target specifically only after the conditional activation of the antibody in target tissues with the goal of limiting on-target off-tumor toxicity. ADG126 in combination with pembrolizumab in microsatellite stable colorectal cancer without liver or peritoneal metastases showed positive clinical efficacy in a phase 1b/2 trial with 24% ORR and 8.5-month mPFS [76]. ADG126 is currently being evaluated in combination with atezolizumab and bevacizumab in a phase 1b/2 study in first-line HCC [77] (Table 2).

Beyond CTLA4, GDF15 has emerged as a novel, potent immunosuppressant in the TME of HCC and therapeutic target for Treg modulation. Interim data from a phase 2a study of visugromab, anti-GDF15 mAb, plus nivolumab in patients with advanced stage, anti-PD-(L)1 relapsed/refractory solid tumors (non-small cell lung cancer, urothelial cancer, HCC) showed an ORR among the 16 HCC patients of 18.8%, with two achieving partial response and one complete response [78] (Table 2). Given the pleiotropic actions of GDF15 with metabolic and immunomodulatory effects, visugromab also has the potential to alleviate treatment-related side effects due to the treatment-induced expression of GDF15, including nausea/vomiting, appetite, and cachexia [79,80].

**Table 2:** Clinical trials for novel immunotherapy approaches in neoadjuvant, adjuvant, and palliative settings.

NCT # [Acronym]	Phase	Study Drug (MOA)	Control	Response	Ref
ICI-ICI-based combinations **Neoadjuvant**					
NCT05440864	2	tremelimumab (anti-CTLA4) + durvalumab (anti-PD-L1)	none	recruiting	[81]
NCT03682276	1b	ipilimumab (anti-CTLA4) + nivolumab (anti-PD-1)	none	MPR 56%	[82]
NCT05908786	1b/2	atezolizumab (anti-PD-L1) + bevacizumab (anti-VEGF-A) +/− tiragolumab (anti-TIGIT) bevacizumab (anti-VEGF-A) + tobemstomig (PD-1/LAG-3 bsAb)	none	recruiting	[83]
NCT04658147	1	nivolumab (anti-PD-1) +/− relatlimab (anti-LAG-3)	none	recruiting	[84]
**Palliative–1L**					
NCT04524871 [MORPHEUS-LIVER]	1/2	tiragolumab (anti-TIGIT) + atezolizumab (anti-PD-L1) + bevacizumab (anti-VEGF-A)	none	ORR 42.5%	[85]
NCT05904886 [IMBRAVE 152]	3		placebo	recruiting	[86]
NCT03680508	2	cobolimab (anti-TIM3) + dostarlimab (anti-PD-1)	none	ORR 46.0%	[87]
NCT04050462	2	BMS986253 (anti-IL8 mAb) or cabiralizumab (anti-CSF1R mAb) + nivolumab (anti-PD-1)	nivolumab	ongoing	[88]
NCT04524871 [MORPHEUS-LIVER]	1b/2	ADG-126 (masked anti-CTLA4) + atezolizumab (anti-PD-L1) + bevacizumab (anti-VEGF-A)	none	ongoing	[77]
NCT04524871 [MORPHEUS-LIVER]	1/2	tobemstomig (PD-1/LAG3 bsAb) + atezolizumab (anti-PD-L1) + bevacizumab (anti-VEGF-A)	none	ongoing	[77]
NCT04524871 [MORPHEUS-LIVER]	1/2	IO-108 (anti-LILRB2) + atezolizumab (anti-PD-L1) + bevacizumab (anti-VEGF-A)	none	ongoing	[77]
NCT04212221	1/2	tebotelimab (PD-1/LAG-3 bsAb)	none	ORR 13.3%	[89]
**Palliative–2L**					
NCT05724563	2	domvanalimab (anti-TIGIT) + zimberelimab (anti-PD-1)	none	ORR 17.2%	[90]
NCT04212221	1/2	tebotelimab (PD-1/LAG3 bsAb)	none	ORR 3.3%	[89]
ICI-targeted therapy combinations **Neoadjuvant**					
NCT04521153	2/3	camrelizumab (anti-PD-1) + apatinib (anti-VEGFR2)	none	MPR 46%	[91]
NCT04954339 [DYNAMIC]	2	atezolizumab (anti-PD-L1) + bevacizumab (anti-VEGF-A)	none	recruiting	[92]
NCT05389527 [Neo-LEAP-HCC]	2	pembrolizumab (anti-PD-1) + lenvatinib (TKI)	none	MPR 38%	[93]
NCT04615143	2	tislelizumab (anti-PD-1) +/− lenvatinib (TKI)	none	ongoing	[94]
NCT03299946	1b	cabozantinib (TKI) + nivolumab (anti-PD-1)	none	MPR 42%	[95]
**Adjuvant**					
NCT04102098 [IMBRAVE 050]	3	atezolizumab (anti-PD-L1) + bevacizumab (anti-VEGF-A)	surveillance	RFS 33.2 mo (36.0 mo with surveillance)	[96]
NCT06059885	2	tislelizumab (anti-PD-1) + TKI	surveillance	recruiting	[97]
**Palliative–1L**					
NCT04524871 [MORPHEUS-LIVER]	1/2	TPST1120 (PPARα inhibitor) + atezolizumab (anti-PD-L1) + bevacizumab (anti-VEGF-A)	none	ORR 30.0%	[49]
NCT06680258	3		placebo	recruiting	[98]
NCT03519997	2	bavituximab (anti-phosphatidylserine mAb) + pembrolizumab (anti-PD-1)	none	ORR 32.1%	[99]
NCT04172571	2	anlotinib (TKI) + penpulimab (anti-PD-1)	none	ORR 31.0%	[100]
ChiCTR1900028295	2	anlotinib (TKI) + toripalimab (anti-PD-1)	none	ORR 29.0%	[101]
NCT04194801	1b/2	fisgatinib (FGFR4 inhibitor) + CS1001 (anti-PD-L1)	none	ORR 50.0%	[102]
NCT04444167	1b/2	cadonilimab (PD-1/CTLA4 bsAb) + lenvatinib (TKI)	none	ORR 35.7%	[103]
NCT03893695	1b/2	ascrinvacumab (anti-ALK1) + nivolumab (anti-PD-1)	none	ORR 30.0%	[67]
NCT02675946 [KEYNOTE 596]	1	CGX1321 (PORCN inhibitor) + pembrolizumab (anti-PD-1)	none	ongoing	[104]
NCT03833700	1	E7386 (CBP–β catenin interaction inhibitor)	none	ongoing	[105]
NCT05022927	1	ERY974 (GPC3/CD3 bsAb) + atezolizumab (anti-PD-L1) + bevacizumab (anti-VEGF-A)	none	ongoing	[106]
--	1-ready	BC2027 (GPC3 ADC)	none	pending	[107]
--	IND-enabling	ZW251 (GPC3 ADC)	none	pending	[108]
**Palliative–2L**					
NCT06361758	2	cadonilimab (PD-1/CTLA4 bsAb) + lenvatinib (TKI)	none	ongoing	[109]
NCT04725474 [GDFATHER]	1/2a	visugromab (anti-GD15) + nivolumab (anti-PD-1)	none	ORR 18.8%	[78]
NCT05091346	1b/2	E7386 (CBP–β catenin interaction inhibitor) + pembrolizumab (anti-PD-1) +/− lenvatinib (TKI)	none	ongoing	[110]
not registered in U.S./EU	1/2	NY-303 (GPC3/NKp46)	none	ongoing	[111]
ICI-Locoregional Therapy combinations **Neoadjuvant**					
NCT05250843	2/3	lenvatinib (TKI) + sintilimab (anti-PD-1) + TACE/HAI	none	recruiting	[112]
NCT05171335	2	lenvatinib (TKI) + TACE	none	recruiting	[113]
NCT05042336	1b/2	camrelizumab (anti-PD-1) + lenvatinib (TKI) + TACE	none	MPR 44%	[114]
**Palliative**					
NCT03778957 [EMERALD-1]	3	durvalumab (anti-PD-L1) + bevacizumab (anti-VEGF-A) + TACE	TACE	ORR 43.6%	[115]
NCT04246177 [LEAP-012]	3	pembrolizumab (anti-PD-1) + lenvatinib (TKI) + TACE	TACE	ORR 46.8%	[116]
NCT06371157	3	cadonilimab (PD-1/CTLA4 bsAb) + lenvatinib (TKI) + TACE	TACE	ongoing	[117]
NCT04340193 [CHECKMATE 74W]	3	nivolumab (anti-PD-1) +/− ipilimumab (anti-CTLA4) + TACE	TACE	ongoing	[118]
NCT04559607	3	camrelizumab (anti-PD-1) + apatinib (anti-VEGFR2) + TACE	TACE	ongoing	[119]
NCT04712643 [TALENTACE]	3	atezolizumab (anti-PD-L1) + bevacizumab (anti-VEGF-A) + TACE	TACE	ongoing	[120]
NCT04268888 [TACE-3]	2/3	nivolumab (anti-PD-1) + TACE	TACE	recruiting	[121]
NCT03572582 [IMMUTACE]	2	nivolumab (anti-PD-1) + TACE	TACE	ORR 71.4%	[122]
NCT06040099 [EMERALD Y90]	2	durvalumab (anti-PD-L1) + bevacizumab (anti-VEGF-A) + TARE	TARE	recruiting	[123]
NCT04541173	2	atezolizumab (anti-PD-L1) + bevacizumab (anti-VEGF-A) + TARE	TARE	terminated due to slow recruitment	[124]
NCT04522544	2	durvalumab (anti-PD-L1) + tremelimumab (anti-CTLA4) + Y90 SIRT or TACE	Y90 SIRT TACE	recruiting	[125]
NCT04663035	2	tislelizumab (anti-PD-1) + ablation	ablation	recruiting	[126]
NCT04150744	2	carrizumab (anti-PD-1) + ablation	ablation	recruiting	[127]
Vaccines/adoptive cell therapy **Palliative–2L HCC**					
NCT04251117	1/2	GNOS-PV02 (personalized therapeutic cancer vaccine) + pembrolizumab (anti-PD-1)	none	ORR 30.6%	[128]
NCT02541370	2	CD133 auto CAR-T	none	ORR 4.8%	[129]
NCT02395250 NCT03146234	1	GPC3 auto CAR-T	none	ORR 15.0%	[130]
NCT05155189	1	C-CAR031 (GPC3 armored auto CAR-T)	none	ORR 50.0%	[131]
NCT06478693	1	MT-303 (GPC3-FcA-LNP CAR)	none	ongoing	[132]
--	IND-ready	in vivo GPC3 CAR-macrophage	none	recruiting	[133]

MPR—major pathologic response, ORR—objective response rate, RFS—recurrence free survival, SIRT—selective internal radiation therapy, TACE—transarterial chemoembolization, TARE—transarterial radioembolization.

Lymphocyte activation gene-3 (LAG-3), T cell immunoglobulin and mucin-domain-containing-3 (TIM-3), and T cell immunoreceptor with immunoglobulin and tyrosine-based inhibitory motif domain (TIGIT) constitute the second wave of immune checkpoint protein targets involved in T and NK cell exhaustion with potential promise for use in the treatment of solid tumors (Figure 1(1)). MORPHEUS-LIVER is a phase 1b/2 open label, multicenter randomized umbrella study assessing atezolizumab and bevacizumab in combination with various investigational therapies in patients with unresectable HCC and no prior systemic therapy—one of those cohorts involves a combination with tiragolumab, an anti-TIGIT mAb. In comparison to doublet therapy with atezolizumab and bevacizumab, the triplet approach yielded a higher ORR (42.5% versus 11.1%) and longer mPFS (11.1 months versus 4.2 months) with no new safety signals, although there was a significant increase in immune-mediated rash (47.5% versus 33.3%) and hypothyroidism (10.0% versus 5.6%) with the addition of tiragolumab [85]. A phase 3 trial is currently underway [86]. Interim results from a phase 2 investigator-initiated study of domvanalimab, a Fc-silent anti-TIGIT mAb, and zimberelimab, an anti-PD-1 mAb, in patients with anti-PD-1/PD-L1 refractory HCC showed that among the 29 evaluable patients, the confirmed ORR was 17.2%, including one complete response, and mPFS was 4.4 months [90]. Cobolimab, an anti-TIM3 mAb, in combination with dostarlimab, an anti-PD-1 mAb, led to an ORR of 46% among 16 evaluable patients with untreated HCC in an ongoing phase 2 study [87] (Table 2).

The development of bispecific antibodies has garnered increasing interest in the past decade as it enables increased specificity and therapeutic applications that cannot be achieved with monoclonal antibodies. There are several bispecific antibodies in development that combine PD-1 inhibition with another immune checkpoint. In the phase 1b/2 COMPASSION-08 trial, cadonilimab, a humanized bispecific antibody (bsAb) targeting PD-1 and CTLA-4, combined with lenvatinib, a multi-tyrosine kinase receptor inhibitor, when given at a higher dose in three-week intervals (cohort B) in the first-line setting, was found to result in an ORR of 35.7%, an mPFS 9.8 months, and an mOS that was not reached (median follow up was 27.4 months) [103]. This combination has been moved forward in a phase 2 study in the second-line setting and in a phase 3 study in combination with TACE in the first-line setting (Table 2). Tebotelimab, a PD-1/LAG-3 bsAb, demonstrated modest antitumor activity, mainly as disease stabilization, in a phase 2 trial in HCC patients with and without prior exposure to ICI. In the ICI-experienced cohort, there was a 3.3% ORR and 50.0% DCR, whereas in the ICI-naïve cohort, there was a 13.3% ORR and 46.7% DCR. mPFS was 2.4 and 3.1 months for ICI-experienced and ICI-naïve cohorts, respectively [89]. Tobemstomig is another PD-1/LAG-3 bsAb that is currently in a phase 1b/2 study under the MORPHEUS-LIVER program, evaluating novel combinations with atezolizumab and bevacizumab in first-line HCC [77] (Table 2). 

The innate immune system has also come into view in recent years as a potential target that could work synergistically with ICIs. For example, a phase 2 trial is underway, assessing nivolumab in combination with BMS-986253, an anti-IL-8 mAb, or cabiralizumab, an anti-CSF1R mAb, both of which target TAMs, either in terms of dampening their pro-tumor function or in reducing their numbers in the TME, respectively [88]. Moreover, another planned cohort in the MORPHEUS-LIVER umbrella study is the combination of IO-108, a fully human IgG4 mAb with high affinity and specificity towards myeloid checkpoint marker LILRB2, with atezolizumab and bevacizumab [77]. Clinical data from the phase 1 dose escalation study of IO-108 as a monotherapy and in combination with pembrolizumab among patients with relapse/refractory solid tumors demonstrated a favorable safety profile and encouraging clinical benefit with an ORR of 9.1% and 23.1%, respectively [134]. Palmitoyl-protein thioesterase (PPT1), an enzyme involved in lysosomal degradation, is mainly expressed in macrophages in HCC, and PPT1+ macrophage infiltration is associated with poor prognosis in HCC [135,136]. Its blockade has been shown to inhibit autophagy in cancer, and when used in combination with PD-1 inhibition in a mouse model of HCC, decreased tumor burden by inducing the penetration of lymphocytes [137]. In a phase 1b study in patients with primary or secondary liver tumors, the PPT1 inhibitor ezurpimtrostat demonstrated a favorable safety profile, exposure, and preliminary signal of activity, i.e., reduction expression of PPT1 in tissues after administration of ezurpimtrostat [138]. A phase 2b ABE-LIVER clinical trial using ezurpimtrostat in combination with atezolizumab plus bevacizumab had been previously underway, but was recently terminated due to recruitment issues. Finally, bavituximab, an anti-phosphatidylserine mAb, has been found in preclinical trials [139] to have pleiotropic pro-inflammatory and -immune stimulating effects on tumor cells, immune cells, and stroma, suggesting that it may augment the activity of ICI; in a phase 2 study in combination with pembrolizumab in the frontline setting, among 28 evaluable patients, the ORR was 32.1% and the mPFS was 6.3 months [99] (Table 2).

#### 4.1.2. Tumor Microenvironment Modulation

Metabolic reprogramming can aid in the evasion of immune destruction, thus promoting resistance to therapy. Targeting metabolic changes, such as glycolysis, fatty acid oxidation, and amino acid metabolism, could improve the efficacy of ICB by reversing the nutrient deficiency present due to the competition between tumor cells and immune cells, and that limits the expansion and effector functions of T cells. Despite demonstrating antitumor activity and synergistic tumor reduction in xenograft and syngeneic murine models when combined with anti-PD-1 therapy [140], the PPARα inhibitor TPST-1120 in combination with nivolumab in a first-in-human phase 1 study across multiple solid tumor types did not yield positive results in three of four patients with HCC experiencing progression of disease [48]. However, in a phase 1b/2 study under the MORPHEUS-LIVER program evaluating TPST-1120 in combination with standard-of-care atezolizumab and bevacizumab in the first-line setting, the triplet led to an improvement across all endpoints: 7-month mPFS versus 4.3 months, 21-month mOS versus 15 months, and 30% ORR versus 13% [49]. A phase 3 study is planned for 2025 (Table 2). While not yet in clinical trials, adenosine A2a receptor (A2aR) inhibition has been combined with anti-PD-1 therapy, leading to the activation of T cells and reductions in tumor size in orthotopic murine liver cancer models [141]. Conversely, Allard et al. demonstrated in murine models that the deletion of the adenosine A2A receptor triggers steatohepatitis and systemic inflammation, leading to spontaneous HCC, thus caution should be exercised in utilizing adenosine blockades in MASH-related HCC [142].

#### 4.1.3. Wnt/β-Catenin Inhibition

Despite the fundamental role of the Wnt/β-catenin pathway in most advanced gastrointestinal cancers like HCC and in mediating resistance to immunotherapy, there are no approved therapies targeting this pathway. One possible therapeutic target within the Wnt/β-catenin canonical pathway is the interaction between CREB-binding protein (CBP) and β-catenin. In mouse mammary tumors treated with E7386, an inhibitor of the interaction between CBP and β-catenin, there was demonstrated downregulation in genes related to hypoxia and increased infiltration of CD8+ T cells into tumor tissues; similar in vivo antitumor activity was seen when E7386 was combined with an anti-PD-1 mAb [143]. E7386 is currently being investigated in a phase 1 study in patients with advanced gastrointestinal tumors [105]. Inhibiting the enzymatic activity of Porcupine (PORCN), an endoplasmic reticulum enzyme that controls the secretion of Wnt, offers an approach that overcomes the limitations of β-catenin inhibitors, which target the canonical pathway, and anti-Frizzled antibodies, which target the non-canonical pathway. Preclinical studies of PORCN inhibitors have demonstrated remarkable efficacy in colorectal cancer, especially those with high Wnt-ligand dependency, defined as those with RNF43 gene mutations and fusions in the RSPO gene family [144]; it remains to be seen how this translates to HCC as well as in clinical studies. CGX1321, a PORCN inhibitor, is currently in a phase 1 trial as a monotherapy in patients with advanced GI tumors, including HCC, with a roll-over cohort in combination with pembrolizumab in patients that have progressed on single-agent CGX1321 [104] (Table 2).

#### 4.1.4. Slowing Angiogenesis

Anti-angiogenic treatment remodels the immune microenvironment by normalizing pro-inflammatory cytokines, activating antigen-presenting cells, polarizing tumor-associated macrophages, and enhancing T cell trafficking [145]. Combining anti-angiogenic drugs with immune-checkpoint inhibitors (ICIs) alters the tumor endothelium, thereby improving drug delivery and increasing the infiltration of effector immune cells [17,69]. Though there has been mixed data to date, with the aforementioned COSMIC-312 study assessing atezolizumab plus cabozantinib [7] and the CARES-310 study evaluating camrelizumab plus rivoceranib [8], there remain several active studies combining ICI with anti-angiogenic treatments. As a single agent, anlotinib, a multi-targeting tyrosine kinase inhibitor, was able to achieve stable disease in most patients with or without prior TKI therapy [146], but when in combination with anti-PD-1 mAbs like penpulimab [100] or toripalimab [101] in the first-line setting, they are able to achieve ORRs of 31% and 29%, respectively. Among the 20 patients enrolled with disease refractory to systemic therapy with sorafenib or lenvatinib who had received ascrinvacumab, an anti-ALK-1 mAb that functions to dampen the TGFβ signaling network, and nivolumab, the ORR was 30% (all partial response), with 60% of patients experiencing progression of disease [67] (Table 2). Fisgatinib, an anti-FGFR4 inhibitor, demonstrated a degree of monotherapy activity, giving evidence of radiographic tumor reductions in 41% of FGF IHC-positive patients, with the majority having received prior TKI and with approximately one-third having received prior ICI [70]; however, as shown in a small study of four patients with FGF19+ HCC and no prior systemic therapy, in combination with CS1001, an anti-PD-L1 mAb, half the patients had a clinical response with a DCR of 100% [102]. There are several ongoing clinical trials for PD-1-VEGF drug conjugates, including PD-1/VEGF bsAbs ivonescimab, which showed promising efficacy signals in multiple advanced, relapsed-refractory solid tumors in a phase 1a study [147], and AK112, which is being evaluated in a phase 2 study in first-line unresectable HCC [148]. In a murine model of HCC, Liu et al. demonstrated that the combination of a VEGFR-targeting peptide-drug conjugate with an anti-PD-1 mAb led to significant tumor growth inhibition and prolonged murine survival [149].

#### 4.1.5. GPC3 Targeting

GPC3 is a well-known TAA in HCC that is linked to poor prognosis. Thus, it has been the target of drug development for over decade via various modalities. The first anti-GPC3 mAb, codrituzumab, showed preclinical antitumor activity by antibody-dependent cell-mediated cytotoxicity, but failed in clinical trials for advanced HCC [150]. Researchers found that cross-linking T cells with GPC3-positive tumor cells with a GPC3/CD3 bsAb mediated potent GPC3-dependent and concentration-dependent cytotoxicity in vitro and significantly inhibited tumor growth in murine xenograft models [151]. SAR444200 is a novel nanobody-based bispecific T cell engager targeting CD3 and GPC3 currently in an open-label phase 1/2 trial with no dose limiting toxicities reported thus far [152]. There is an ongoing phase 1 study evaluating GPC3/CD3 bsAb ERY974 in combination with atezolizumab and bevacizumab in patients with locally advanced or metastatic HCC [106]. Another bispecific combination that has been assessed in vivo is one targeting GPC3 and CD47, an innate immune checkpoint marker that promotes the evasion of tumors from immune surveillance by acting as the well-established “don’t-eat-me” signal. This bispecific antibody was found to mediate strong effector functions via macrophages and neutrophils against dual antigen-expressing HCC cells. It outperformed both monotherapies and a combination therapy with anti-CD47 and anti-GPC3 mAbs in a xenograft HCC model [153]. NY-303 is another bispecific antibody targeting GPC3 and NKp46, a cell surface receptor on natural killer cells, that is able to shift the TME into an immunologically active milieu that is more susceptible to ICI. Preclinical in vitro and in vivo models have shown that NY-303 is highly potent and effective in mediating NK cell-redirected cytotoxicity against multiple HCC tumor cell lines [154] and in down-modulating GPC3 expression and inhibiting beta-catenin activation, resulting in a PD-1 checkpoint blockade-sensitive biomarker signature [111]. A phase 1/2 study assessing NY-303 in patients not responding to first-line immunotherapy is currently ongoing (Table 2).

Antibody-drug conjugates (ADCs) combine the selectivity of antibodies with the efficacy of highly potent chemotherapies to efficiently kill tumor cells while attempting to spare normal tissue. There are several approved ADCs for hematologic malignancies as well as breast and head and neck cancers. Research in ADCs has also extended into payload diversification going beyond topoisomerase 1 inhibitors to include immune stimulants (e.g., TLR7/8 agonists), kinase inhibitors, and proteolysis-targeting chimeras (PROTACs) [155]. There are a few experimental preclinical and clinical approaches underway for an ADC-based treatment of HCC targeting B7-H3 [156], CD147 [157], and CD24 [158] among others. Notably, the ability of GPC3 to internalize enables the use of ADCs. In designing an effective GPC3-targeted ADC, Fu et al. screened over 9000 compounds against HCC cell lines and identified DNA-alkylating agents, duocarmycin SA and pyrrolobenzodiazepine dimer, as the most potent payloads to construct two GPC3-specific ADCs, hYP7-DC and hYP7-PC, respectively. hYP7-PC was found to have single-agent efficacy in multiple murine models. Gemcitabine was found to have a synergistic effect with hYP7-DC both in vitro and in vivo [159]. Madera et al. developed another ADC targeting GPC3 composed of a humanized IgG1 antibody conjugated to a topoisomerase 1 inhibitor, ZW251, with evidence of potent and target-specific cytotoxicity in a panel of HCC cells, as well as robust tumor growth inhibition after a single administration in several cell-line derived and patient-derived xenograft murine models representing a wide-range of GPC3 expression [108]. ZW251 is currently being used in IND-enabling studies. BC2027 is a first-in-class ADC targeting GPC3 that, as of April 2024, received FDA clearance for in-human studies in GPC3-positive cancers after demonstrating in preclinical studies greater than 90% inhibition of tumor growth in some well-established GPC3-positive cancers [107] (Table 2).

#### 4.1.6. Utilizing Locoregional Therapy

There is an increasing body of evidence to suggest that locoregional therapies (LRTs), such as transarterial chemoembolization (TACE) and transarterial radioembolization (TARE), can potentiate tumor immunity and enhance the effects of ICIs. TACE can create a pro-inflammatory TME via local tumor necrosis which facilitates the release of tumor neoantigens and leads to the recruitment and activation of dendritic cells [160,161]. There are several trials that explore the combination of LRT with ICIs and/or TKIs. EMERALD-01 is a phase 3 study in patients with embolization-eligible unresectable HCC who were randomized to TACE + durvalumab + bevacizumab (D+B+TACE) or TACE + durvalumab (D+TACE), or TACE alone. Patients treated with D+B+TACE were found to have a statistically significantly greater ORR and longer mPFS compared to TACE alone (43.6% versus 29.6% and 15.0 months versus 8.2 months, respectively). Patients treated with D+TACE had a comparable ORR and mPFS to D+B+TACE (41% and 10 months, respectively). In the D+B+TACE, D+TACE, and TACE-alone cohorts, grade 3+ treatment-related adverse events (TRAEs) occurred in 32.5%, 15.1%, and 13.5% of patients, respectively [115] (Table 2). EMERALD-Y90 is a phase 2 study similar in design to EMERALD-01, but utilizing TARE rather than TACE, with interim results pending [123]. Like EMERALD-01, LEAP-012 is a phase 3 study in a similar population of patients evaluating TACE + pembrolizumab + lenvatinib versus TACE alone [162]. Interim results after a median 25.6-month follow up showed statistically significant and clinically meaningful improvement in progression-free survival at 14.6 months with TACE + pembrolizumab + lenvatinib (P+L+TACE) versus 10.0 months with TACE alone. However, grade 3+ TRAEs occurred in 71.3% of patients treated with P+L+TACE versus 31.5% with TACE alone [116] (Table 2). There are several active clinical trials evaluating LRT in conjunction with ICI-ICI- or ICI-TKI-based combinations in the neoadjuvant, adjuvant, and palliative settings (Table 2). At this time, until an overall survival benefit is demonstrated, the main utilization of LRT-ICI combinations appears to be for the purpose of downstaging to increase the chance of subsequent definitive intervention (i.e., resection, transplant). Moreover, the sequential use of LRT followed by ICI versus concurrent start is an area where further clinical research is needed.

### 4.2. Vaccines/Adoptive Cell Therapy

The role of cancer vaccines in HCC is to reshape the TME and foster durable immune memory. There are various categories of cancer vaccines, including those peptide-based, dendritic-cell-based, viral vector-based, and DNA/mRNA-based [163]. Peptide-based vaccines targeting GPC3 have undergone phase 1/2 clinical trials that achieved predominantly stable disease in patients [164]. However, a promising approach involves DNA plasmid personalized therapeutic cancer vaccine (PTCV) encoding up to 40 neoantigens co-administered with plasmid-encoded IL-12 and pembrolizumab. In a phase 1/2 trial involving TKI-experienced but ICI-naïve patients, this regimen led to an ORR of 30.6% (11/36), with 3/36 patients achieving complete response [128], compared to 21% ORR with nivolumab monotherapy [165] and ORR 17% with pembrolizumab monotherapy [166] in a similar patient population. Further studies demonstrated a higher frequency and number of T cell clones present in the peripheral blood and tumor after vaccination, and a more cytotoxic phenotype among PTCV-expanded T cells [128] (Table 2). 

Adoptive cell therapy, e.g., chimeric antigen receptor (CAR) T cell therapy, has become a key strategy in the treatment of advanced hematologic malignancies; however, its use in solid tumors has been mired by the immunosuppressive TME characteristic of many solid tumors, including HCC. Given one of the drivers of ICI resistance is the persistence of LCSCs, a CD-133-directed CAR T cell therapy was developed and assessed in a single arm, open label phase 2 trial in patients who had experienced disease progression after at least one systemic therapy, namely sorafenib. Among the 21 evaluable patients, 16 had previously received sorafenib, and among those patients, the therapy was found to yield stable disease in the majority of patients (9/16), with the best response being a partial response, 6.8-month mPFS, and 12-month mOS [129]. A phase 1 trial of a CAR T cell therapy targeting GPC3 in patients with HCC that have progressed following surgery, locoregional therapy, or systemic therapy did not yield results similar to those of other modalities targeting GPC3, with the majority of patients experiencing progression of disease leading to an ORR of 15%; among the two patients that received prior sorafenib, one experienced stable disease, and the other progression [130]. Li et al. constructed bispecific GPC3 and PD-1 CAR T cells that demonstrated sustained toxicity against PD-L1+ HCC cells in vitro and induced tumor suppression, and extended survival in PD-L1+ HCC xenograft murine models, as opposed to their single-target counterparts [167]. It remains to be seen how this translates in first-in-human studies. C-CAR031 is an autologous GPC3-directed CAR-T armored with dominant negative TGFβR2 that, in a phase 1 study with 24 patients that had been previously treated with systemic therapy, including ICI, demonstrated tumor reductions in 91% of patients, including a median reduction of 44% of extrahepatic lesions, and an ORR of 50% [131]. A different emerging adoptive cell therapy approach is programming myeloid cells rather than T cells to express GPC3- targeting CAR. Preclinical studies of MT-303, a novel LNP-formulated GPC3-specific CAR mRNA for the in vivo programming of myeloid cells, has demonstrated strong expression in myeloid cells as well as antitumor activity with increased cytokine and chemokine levels in peripheral blood [168]. A phase 1 study of MT-303 is currently enrolling. Another macrophage-targeted in vivo therapy against GPC3 using mRNA/LNP technology developed by Carisma Therapeutics and Moderna has also demonstrated a similar favorable preclinical profile with potent dose-dependent cytotoxicity against GPC3+ tumor cells [133] (Table 2).

### 4.3. Novel Timing–Moving Immunotherapy to the Perioperative Setting

To date, most of the aforementioned research regarding ICI-based therapies has been in the context of advanced, unresectable HCC. Given that ICI antitumor response hinges on interactions among T cells, antigen-presenting cells, and tumor cells, and such interactions are likely to occur when there is a large tumor burden presented with the primary tumor, and to a certain extent with micrometastases, there is a potential mechanistic rationale for neoadjuvant and adjuvant use of ICI in HCC [169]. Thus, there is an opportunity to move ICI to the neoadjuvant and adjuvant settings for resectable disease, and this has become an area of active investigation. In a cross-trial, patient-level analysis of 104 patients treated with neoadjuvant ICI before liver resection, a major pathological response was observed in 33 patients (32%) and a pathological complete response in 19 patients (18%). After a median follow up of 27.2 months, median recurrence-free survival (RFS) was significantly longer in patients with major pathological response than in those who did not have a major pathological response (not reached [95% CI not evaluable–not evaluable] versus 28.3 months [12.8–43.8]; hazard ratio 0.26 [0.10–0.66]; *p* = 0.0024) and in patients with pathological complete response than in those who did not have a pathological complete response (not reached [95% CI not evaluable–not evaluable] versus 32.8 months [15.0–50.5]; 0.19 [0.05–0.78]; *p* = 0.010). The threshold of 90% tumor regression was found to be the optimal cutoff of pathological tumor regression to predict improved relapse-free survival [170]. In a separate cohort, a single-center analysis of 92 patients that underwent surgical resection for HCC, 36 received neoadjuvant ICI-based therapy, and of those, 61% were outside of standard respectability criteria and were more likely to have features known to confer risk of disease recurrence. Patients who received neoadjuvant ICI had similar rates of margin-negative resection (*p* = 0.47) and recurrence-free survival as those who underwent upfront surgical resection (median RFS 44.8 months compared with 49.3 months, respectively) [171]. In the adjuvant setting, the initial interim analysis from the IMBRAVE050 trial appeared to demonstrate significant improvements in RFS with adjuvant atezolizumab plus bevacizumab compared to active surveillance in high-risk patients after surgical resection or local ablation (median RFS (95% CI): NE (22.1-NE) [atezolizumab plus bevacizumab] versus NE (21.4-NE) [active surveillance], hazard ratio 0.72 (0.53–0.98)) [172,173]. In that analysis, those that underwent resection with an initial tumor size >5 cm derived the greatest benefit in terms of RFS with adjuvant atezolizumab plus bevacizumab (median RFS (95% CI): 19.4 months [atezolizumab plus bevacizumab] versus 14.0 months [active surveillance], hazard ratio 0.66 (0.48–0.91)) [172]. Disappointedly, this RFS benefit was not sustained in an updated survival follow up (the hazard ratio was 0.90 with a 95% CI 0.72–1.12) [96] (Table 2). To date, an OS benefit of adjuvant systemic therapy over standard post-operative surveillance has remained elusive. 

## 5. Summary and Future Directions

HCC is a highly aggressive malignancy whose treatment landscape has significantly changed in the past decade to include immunotherapies, targeted therapies, and combination treatments. The phase III IMBRAVE150 trial that assessed the combination of atezolizumab plus bevacizumab provided proof of concept that tackling both the immune system and angiogenesis would yield superior clinical benefits over then current standard of care, sorafenib. Despite these advances, most patients have a limited response to ICI-based therapy.

In this review, we explored mechanisms of resistance to ICI and proposed approaches to enhance their efficacy. HCC tumors have an immunologically “cold” TME that breeds resistance to ICI, and thus tackling the cellular components, such as TAMs, and non-cellular components, such as metabolic adaptations under hypoxia, that contribute should work synergistically with ICI to improve tumor killing. Tumor heterogeneity, as reflected in the activation of cell signaling pathways, such as Wnt-β-catenin, in the secretion of pro-angiogenic factors, such as VEGF, and in the expression of TAAs, such as GPC3, further promote immune evasion, and have shown preclinical and early clinical evidence of being significant therapeutic targets in combination with ICI. The large tumor burden presented by the primary HCC tumor, and to a certain extent with micrometastases, provides a mechanistic rationale for using ICI in the neoadjuvant and adjuvant settings, respectively. 

An additional key to optimizing modern immunotherapy in HCC will be the use of prospectively validated predictive biomarkers to aid in patient selection and treatment decisions. Clinical informaticists have attempted to develop nomograms for tumor response in patients treated with ICI plus targeted therapy; those to date have identified clinical characteristics, such as tumor size less than 5 cm, higher AFP response (defined as a decline of ≥20% in AFP levels within the initial eight weeks of treatment), fewer extrahepatic metastasis, and lower leukocyte count, as being associated with a higher ORR and PFS [174,175,176]. The development of such predictive models is of particular importance considering the rising incidence of HCC arising in the background of MASH. While patients with HCC due to non-viral etiologies, such as MASH, still do benefit from immunotherapy-based regimens, the effect is not as pronounced as it is in patients with viral etiologies [177]. Therapeutic strategies to target this growing subpopulation of HCC will continue to grow in importance in the coming years. 

Looking forward, the most immediate practice-impacting results will come from reports and data maturation of trials assessing combinatorial locoregional therapy with ICI-based regimens for liver-dominant HCC. In advanced metastatic disease, second-generation immune checkpoint inhibitors (e.g., TIGIT, LAG3, TIM3) added to an ICI and anti-VEGF backbone suggests the future of first-line HCC treatment is moving towards triplet systemic therapy. Finally, utilizing GPC3 as a vehicle for optimal tumor-specific immune targeting is an area of clinical therapeutic development to watch. 

## 6. Conclusions

The treatment landscape for advanced, unresectable and metastatic HCC has significantly evolved in the past decade with immunotherapy-based regimens becoming the foundation of first-line therapy. With less than one third of patients achieving an objective response to immunotherapy-based therapy, we need to understand and address the barriers to ICI response in order to optimize ICI efficacy. Based on our current understanding of mechanisms of resistance to ICI, emerging immunotherapy approaches include second-generation immune checkpoint inhibitors affecting the innate and adaptive immune systems, tumor microenvironment modulators addressing metabolic rewiring under hypoxia, targeted delivery systems directed against pathogenic cell-signaling pathways and tumor-associated antigens, and locoregional interventions. As novel agents and treatment modalities are added to standard ICI-based regimens, minimizing treatment-related adverse events and building effective biomarker-driven strategies that improve patient selection will be key in this ongoing immunotherapeutic revolution in HCC.

## Figures and Tables

**Table 1:** Pivotal trials for current immunotherapy combinations for first-line advanced, unresectable HCC.

NCT # [Acronym]	Year	Study Drug (MOA)	Control	N	mPFS (mo) (Study vs. Control)	mOS (mo) (Study vs. Control)	Ref
NCT03434379 [IMBRAVE 150]	2020	atezolizumab (anti-PD-L1) + bevacizumab (anti-VEGF-A)	sorafenib	501	6.8 vs. 4.3	19.2 vs. 13.4	[1]
NCT03298451 [HIMALAYA]	2022	tremelimumab (anti-CTLA4) + durvalumab (anti-PD-L1) [STRIDE]	sorafenib alone	1171	3.8 vs. 4.1	16.4 vs. 13.8	[3]
NCT03755791 [COSMIC 312]	2022	cabozantinib (TKI) + atezolizumab (anti-PD-L1)	sorafenib alone	837	6.8 vs. 4.2	16.5 vs. 15.5	[7]
NCT03764293 [CARES 310]	2023	camrelizumab (anti-PD-1) + rivoceranib (anti-VEGFR2)	sorafenib	543	5.6 vs. 3.7	22.1 vs. 15.2	[8]
NCT03713593 [LEAP 002]	2023	lenvatinib (TKI) + pembrolizumab (anti-PD-1)	lenvatinib + placebo	794	8.2 vs. 8.0	21.2 vs. 19.0	[6]
NCT04039607 [CHECKMATE 9DW]	2024	nivolumab (anti-PD-1) + ipilimumab (anti-CTLA4)	lenvatinib or sorafenib	668	9.1 vs. 9.2	23.7 vs. 20.6	[11]

## Data Availability

No new data were created or analyzed in this study. Data sharing is not applicable to this article.
